# DSM‐5‐TR Criteria and Domains for Narcissistic Personality Disorder: Evidence From Network Analysis Based on the Mental Health Professionals' Perspective

**DOI:** 10.1002/cpp.70179

**Published:** 2025-11-25

**Authors:** Alessio Gori, Eleonora Topino

**Affiliations:** ^1^ Department of Health Sciences University of Florence Florence Italy; ^2^ Integrated Psychodynamic Psychotherapy Institute (IPPI) Florence Italy; ^3^ Department of Human and Social Sciences Mercatorum University Rome Italy

**Keywords:** antagonism, attention seeking, DSM‐5 diagnosis, narcissistic personality disorder, need for admiration, network analysis, pathological narcissism, personality disorder

## Abstract

Narcissistic personality disorder (NPD) is a complex disorder that, given the variety of its manifestations and the significant challenges related to its treatment, has attracted considerable attention from international scientific research. The present research aimed to investigate the centrality and the dynamics among the symptoms of NPD, based on the mental health professionals' perceptions. The research involved 376 mental health professionals, which evaluated the relative importance of diagnostic criteria for NPD outlined in Section II of the DSM‐5‐TR and the significance of maladaptive personality trait domains encompassed by Criterion B of Section III. Network analysis was then employed to analyse the collected data. Analysis of the NPD criteria network revealed two distinct symptom clusters related to the self and interpersonal dimensions of functioning. The need for admiration criterion emerged as a central node within this network. Furthermore, the network analysis concerning the domains confirmed the centrality of the antagonism domain in NPD. The findings of this study systematise the perspectives of mental health professionals using a network analysis approach to deepen our understanding of the core characteristics of NPD. These insights may offer valuable practical applications for both research and clinical practice, enhancing diagnostic accuracy and informing effective treatment strategies.

## Summary

- From mental health professionals' perspectives, need for admiration represents the most central feature within the DSM‐5 criteria network for narcissistic personality disorder, connecting self‐oriented and interpersonal aspects of the disorder.
- Antagonism emerged as the most central feature within the DSM‐5 Section III network of diagnostic domains for narcissistic personality disorder, based on mental health professionals' perspectives.
- Network analysis offers a valuable framework for identifying central elements in personality disorders, supporting more focused clinical interventions.
- Targeting the central nodes of need for admiration and antagonism could represent an effective therapeutic pathway, as changes in these pivotal elements may propagate throughout the network and promote overall clinical improvement.

## 1 Introduction

### 1.1 Narcissistic Personality Disorder

The diagnosis of narcissistic personality disorder (NPD) describes pervasive patterns of grandiosity, a constant need for admiration and a lack of empathy for others. Individuals with NPD often exhibit an inflated sense of self‐importance, fantasies of unlimited success or power and a tendency to exploit interpersonal relationships to achieve their ends (American Psychiatric Association [APA] 2013, 2022). This disorder is included both in Section II of the DSM‐5‐TR (APA 2022) and in Section III within the Alternative Model of Personality Disorders, where it is detailed with two pathological personality traits relating to the domain of antagonism: grandiosity, characterised by feelings of entitlement and exaggerated self‐importance and attention‐seeking, involving behaviours that draw attention to oneself and elicit admiration from others. NPD is associated with an increased risk of comorbid mood disorder (Nagel et al. 2023), addictions (Walter 2016) and other personality disorders (Hörz‐Sagstetter et al. 2018; Burkle 2016). Patients diagnosed with NPD also experience high levels of distress (Kacel et al. 2017), anxiety (Eaton et al. 2017) and suicidal ideation (Ronningstam et al. 2018), as well as significant impairments in relationships, resulting in interpersonal problems (Day et al. 2022). Moreover, perfectionism (Smith et al. 2016), shame (Ritter et al. 2014), and fear (Ronningstam and Baskin‐Sommers 2013) have been frequently associated with NPD. Given its clinical relevance, NPD has gained growing research interest over the last decade (see Weinberg and Ronningstam 2022 for a review). However, its multifaceted nature (Wright and Edershile 2018), which emerges encompassing a spectrum of severity and presentations (e.g., grandiosity and vulnerability), fuels the need for further investigation (Pincus et al. 2014) to support a deeper understanding and inform and refine therapeutic approaches.

### 1.2 Applying the Network Perspective on NPD

Several authors (see Skodol et al. 2014 for a review) criticised the categorical approach of DSM‐5 (APA 2013, 2022), arguing that it describes only the grandiose subtype of the NPD (i.e., overt grandiosity, arrogance and less observable anxiety) and fails to capture the characteristics of the vulnerable subtype (i.e., covert, inhibited, overtly distressed, hypersensitive to others' evaluations while chronically envious and evaluating oneself in relation to others). Other explorations, on the other hand, suggested that the criteria can represent both the subtypes (Fossati et al. 2005). Parallelly, Section III of the DSM‐5‐TR only lists two pathological personality traits under the antagonism domain (APA 2013, 2022), although within the text, it is indicated that traits in other domains could be identified depending on the individual's presentation (grandiose vs. vulnerable). Therefore, given the complexity of the disorder, further investigations are needed. In this regard, network analysis is an innovative approach that can be effectively applied to psychopathology (Borsboom 2017). This approach enables the conceptualisation of the disorder as a network of symptoms, statistically exploring their relational patterns. Moreover, network analysis provides insights into the influence and centrality of each symptom within the framework, thereby identifying the elements that may be relevant in strengthening or weakening the internal symptomatic network (Borsboom and Cramer 2013; Cramer et al. 2010). This approach has already been previously applied to other personality disorders (Marian et al. 2022; Gori et al. 2025). Concerning NPD, network analysis has previously been used to explore the role of conditions and symptoms associated with the disorder (e.g., Abdelrahman et al. 2024; Dinić et al. 2021; Jordan et al. 2022), but there is not yet an application to the criteria and domains of the DSM‐5‐TR (APA 2013, 2022).

### 1.3 The Present Research: The Mental Health Professionals' View on NPD Criteria and Domains

Although NPD has been extensively examined (see Levy and Rosenstein 2020 for an overview), two gaps remain salient. First, the field lacks a systematic account of how clinicians (who integrate theoretical knowledge with cumulative, real‐world observations) perceive and prioritise the DSM‐5‐TR Section II criteria and Section III domains of NPD (APA 2013, 2022). Because personality disorders are often egosyntonic (Hart et al. 2025), self‐report data may underrepresent or misrepresent clinically relevant features, whereas clinicians routinely observe how diagnostic characteristics manifest across settings, interpersonal contexts and phases of treatment (e.g., alliance ruptures, shame reactivity and regulation of self‐esteem). Capturing clinicians' perspectives can therefore clarify which criteria are most salient in practice and refine case formulation and diagnostic decision‐making. Second, there is a need for an analytic approach that formally models the interrelations among NPD features. Network analysis conceptualises disorders as systems of mutually interacting elements rather than as reflections of a single latent cause, estimating conditional associations among nodes (criteria/domains) and quantifying each node's centrality (Borsboom and Cramer 2013; Borsboom 2017; Cramer et al. 2010; Epskamp et al. 2018; Burger et al. 2023). Compared with traditional correlational or latent variable approaches, this framework can identify central or pivotal features that may organise the diagnostic system, reveal clusters of tightly connected symptoms and suggest leverage points where clinical change may cascade through the broader symptomatic network. However, to the authors' knowledge, network analysis has not yet been applied to the DSM‐5‐TR criteria and domains of NPD (APA 2013, 2022) from clinicians' perspectives.

To fill these gaps, the general aim of the present research is to analyse the centrality and the dynamics among the symptoms of NPD, based on the mental health professionals' perception. The specific goals were as follows:

1. To explore the NPD's criteria network, examining the associations and centrality among the nine criteria of NPD described within Section II of the DSM‐5‐TR (APA 2013, 2022).
2. To explore the NPD's domains network, examining the associations and centrality among the five domains included within Section III of the DSM‐5‐TR (APA 2013, 2022).

## 2 Method

### 2.1 Participants, Procedure and Ethics

The study included 376 participants (*M*_*age*_ = 41.42; *SD* = 12.649; 78% females; 22% males). They were all mental health professionals, specifically psychologists (*n* = 134), psychiatrists (*n* = 6) and psychotherapists (*n* = 236). Most of them were married (37%), single (34%), or cohabiting (22%). Among psychotherapists, the most reported theoretical orientations were cognitive behavioural (13%), psychoanalytic (12%), psychodynamic (10%) and systemic (10%). Participants were recruited using a snowball sampling method, where initial contacts provided by the researchers led to referrals from other potential participants. The inclusion criterion was to be a licensed mental health professional. The survey was administered online through the Google Forms platform after giving electronic informed consent. All the procedures of this research were approved by the first author's institutional Ethical Committee.

### 2.2 Measures

#### 2.2.1 Demographics and Professional Background

Participants were asked to provide information about their background (age, gender, marital status) and professional experience (professional qualification, and, for psychotherapists, the theoretical orientation).

#### 2.2.2 NPD Criteria

Based on the DSM‐5‐TR Section II (American Psychiatric Association [APA] 2013, 2022), participants were asked to evaluate the significance of the NPD criteria in its diagnosis, based on their clinical experience. Their responses were on a 5‐point Likert scale, from 1 (*minimal importance*) to 5 (*great importance*).

#### 2.2.3 NPD Domains

Based on the DSM‐5‐TR Section III (American Psychiatric Association [APA] 2013, 2022), participants were asked to evaluate the representativeness of each domain for NPD, based on their clinical experience. Their responses were on a 5‐point Likert scale, from 1 (*not at all representative*) to 5 (*very representative*).

### 2.3 Data Analysis

Statistical analysis was conducted using the statistical software program JASP (Jeffrey's Amazing Statistics Program, v. 0.18.3; JASP Team 2023). Two networks were developed: one for the criteria and one for the domains. In performing network analysis, the estimation technique called EBICglasso was employed. This approach leverages the graphical Least Absolute Shrinkage and Selection Operator (LASSO; Friedman et al. 2008) regularisation for model selection, informed by the Extended Bayesian Information Criterion (EBIC; Chen and Chen 2008). A tuning parameter of 0.5 was employed to construct a network that is both specific (captures true relationships) and sensitive to subtle connections between the observed variables. These variables are represented by nodes in the network, and the connections between them are called edges (Burger et al. 2023). First, the overall network structure was examined by interpreting the associations (edges) between nodes based on their weights. Following Ferguson's (2016) criteria, edge weights were categorised as follows: ≤ 0.2 (small), > 0.2 to ≤ 0.5 (moderate), and > 0.5 (large). Subsequently, three centrality indices were analysed (Epskamp et al. 2018; Opsahl et al. 2010): (1) Betweenness centrality: This index captures the frequency with which a node acts as a bridge between other nodes on the shortest path connecting them within the network. (2) Closeness centrality: This index reflects how close a node is to all other nodes in the network, indicating its efficiency in information dissemination. (3) Strength centrality: This index quantifies the total number of connections a node has within the network. Case‐dropping bootstrapping (repeated 1000 times) with 95% confidence intervals was employed to assess the network's stability. This technique involves resampling the data with replacement, creating new network estimates for each resample with a reduced number of cases. Coefficients exceeding 0.5 were considered indicative of acceptable stability (Epskamp et al. 2017; Epskamp et al. 2018).

## 3 Results

**FIGURE 1:**
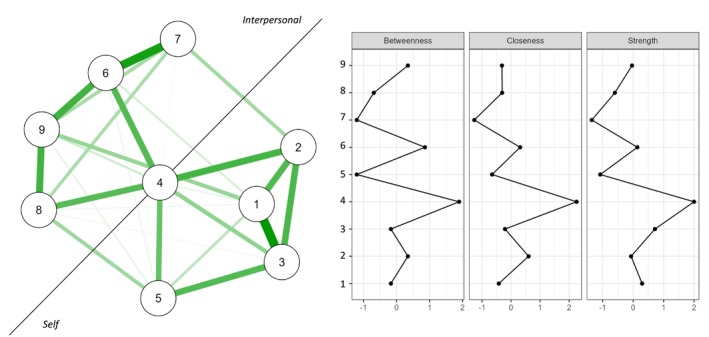
Network analysis for the narcissistic personality disorder criteria. *Note*: Green lines show positive relationships between criteria. Thicker lines indicate stronger edge weights. Criterion 1 = grandiosity; Criterion 2 = fantasies of unlimited success; Criterion 3 = belief of being special; Criterion 4 = need for admiration; Criterion 5 = sense of entitlement; Criterion 6 = exploitation of others; Criterion 7 = lack of empathy; Criterion 8 = envy; Criterion 9 = arrogance.

The network of criteria for NPD consisted of nine nodes, and 30/36 edges were nonzero (see Figure 1). Within the network, edge weights showed two groups: The first included criteria 1, 2, 3, and 5; the second included criteria 6, 7, 8, and 9; Criterion 4 seemed to be a common element. Specifically, Criterion 1 (grandiosity) was associated with Criterion 2 (fantasies of unlimited success; *r =* 0.228) and Criterion 3 (Belief of being special; *r* = 0.331). Criterion 2 (fantasies of unlimited success) related to Criterion 3 (belief of being special; *r =* 0.234) and Criterion 4 (need for admiration; *r =* 0.243). Criterion 3 (belief of being special) was also linked to Criterion 5 (sense of entitlement; *r* = 0.225). Furthermore, Criterion 4 (need for admiration) was associated with Criterion 6 (exploitation of others; *r* = 0.224) and Criterion 8 (envy; *r* = 0.219). Criterion 6 (exploitation of others) was also linked to Criterion 7 (lack of empathy; *r* = 0.306) and Criterion 9 (arrogance; *r* = 0.249). Criterion 9 (arrogance) was also linked to Criterion 8 (envy; *r* = 0.250).

**FIGURE 2:**
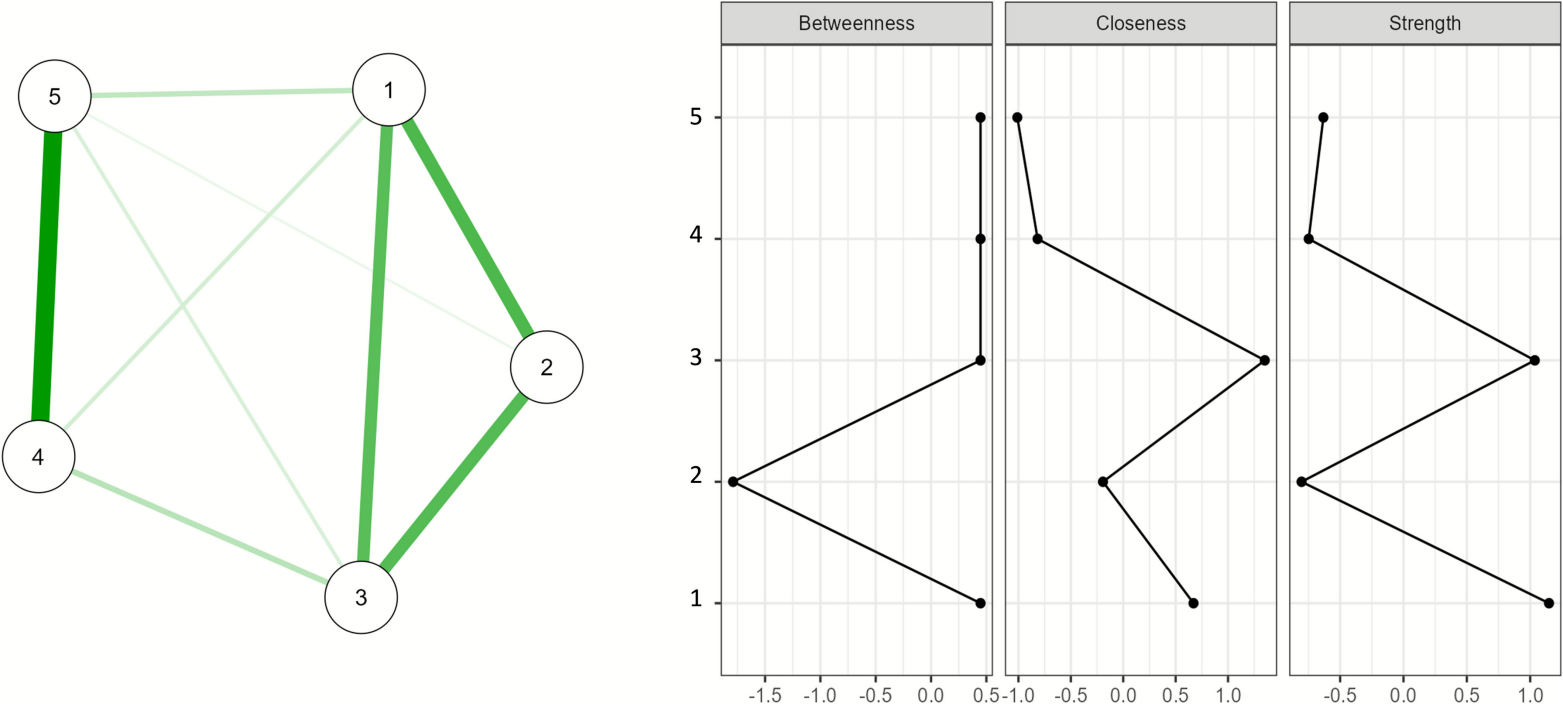
Network analysis for the narcissistic personality disorder domains. *Note*: Green lines show positive relationships between criteria. Thicker lines indicate stronger edge weights. Domain 1 = negative affectivity; Domain 2 = detachment; Domain 3 = antagonism; Domain 4 = disinhibition; Domain 5 = psychoticism.

The network of domains for NPD consisted of five nodes, and 9/10 edges were nonzero (see Figure 2). Within the network, Domain 3 (antagonism) showed the highest number of medium‐range connections, specifically with Domain 1 (negative affectivity; *r* = 0.293) and Domain 2 (detachment; *r* = 0.300). Domain 1 (negative affectivity) was also associated with Domain 2 (detachment; *r* = 0.312). Finally, Domain 4 (disinhibition) was related to Domain 5 (psychoticism): *r* = 0.450.

**FIGURE 3:**
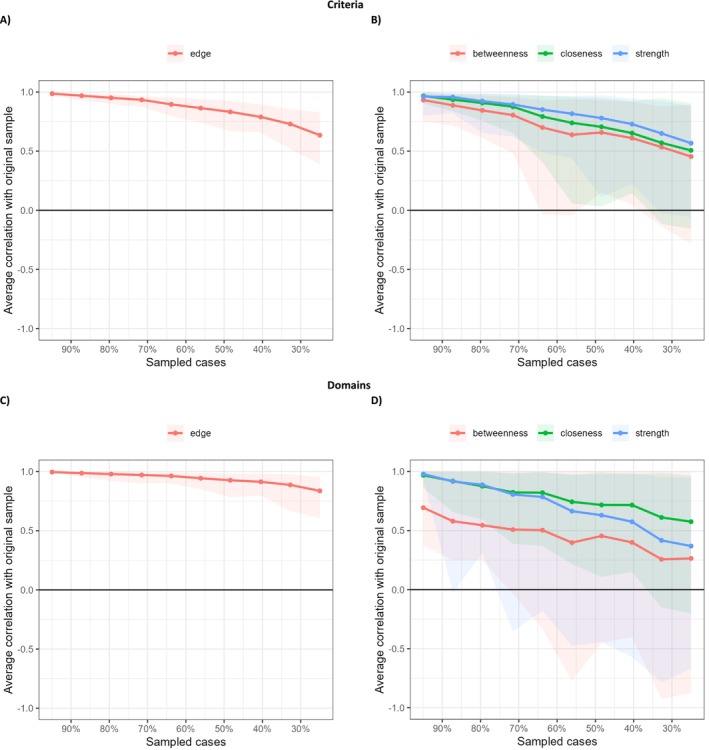
Accuracy of the edge‐weight estimates (Sections A and C) and stability of centrality indices (Sections B and D) based on bootstrap. *Note*: Areas indicate 95% CI.

The bootstrap analysis supported the accuracy of the edge‐weight estimates (see Figure 3A,C).

The indices of centrality were also explored. Concerning the criteria, the bootstrap analysis supported the interpretability of the strength and closeness indexes, since they showed values higher than 0.50 (see Figure 3, part B). Specifically, Criterion 4 (need for admiration) presented the highest level of strength and closeness (see Figure 1).

Concerning the domains, the bootstrap analysis supported the interpretability of the closeness index, with values higher than 0.50 (see Figure 3, part D). Specifically, Domain 3 (antagonism) showed the highest level of closeness (see Figure 2).

## 4 Discussion

NPD is a multifaceted phenomenon that creates significant suffering both in the individuals who suffer from it and for those close to them (see Yakeley 2018 for a review). Given its complexity, much research has paid attention to the DSM‐5‐TR definition with respect to the various manifestations of the phenomenon (Fossati et al. 2017) to provide a better understanding of this condition and guide the diagnosis and treatment processes. Aligned with this framework, the present study sought to investigate the centrality and dynamics among the symptoms of NPD from the perspectives of mental health professionals. To accomplish this, the network analysis approach was utilised. This method offers a two‐pronged benefit: firstly, by visualising the criteria as a network, it exposes potential clusters and patterns within the disorder; secondly, it allows quantification of each criterion's centrality, revealing which holds the most influence. By investigating these network characteristics, valuable insights with the potential to refine diagnostic approaches and inform the development of more effective treatment strategies could be highlighted (see Borsboom and Cramer 2013 for a review).

### 4.1 Self and Interpersonal Clusters in NPD Criteria: The Centrality of Need for Admiration

Explorations of node–node relationships using edge weights highlighted groupings among the criteria (see Figure 1). On the one hand, connections have been observed between the criteria of grandiosity, fantasies of unlimited success, belief in being special, and sense of entitlement. On the other hand, associations have been highlighted between the criteria of exploitation of others, lack of empathy, envy, and arrogance. Furthermore, these two groups have been interconnected through the common association with the need for admiration criterion. These findings appear to mirror the impairment in personality functioning described in Section III of the DSM‐5 (APA 2013, 2022), reflecting alterations in the self (grandiosity, fantasies of unlimited success, belief in being special, and sense of entitlement) and interpersonal dimensions (exploitation of others, lack of empathy, envy, and arrogance). Furthermore, the need for admiration criterion emerges as a common element between the two groupings, highlighting its significance in both self (i.e., need for admiration to regulate self‐esteem and set goals based on seeking approval from others) and interpersonal (i.e., attentiveness to others' reactions perceived as relevant to oneself and exploiting relationships to regulate self‐esteem) functionings. These data differ from previous studies on the criteria, where authors suggested a possible subdivision based on overt or covert manifestations (Fossati et al. 2005). However, they are not contradictory but rather provide further enrichment of the conceptualisations of the narcissism construct at multiple levels of organisation (e.g., overt and covert presentations, trifurcated model; Weiss et al. 2019). Indeed, existing research supports the possibility that individuals with NPD fluctuate between states of grandiosity and vulnerability depending on life circumstances or may even present mixed characteristics (see Caligor et al. 2015 for a review). Therefore, diagnostic criteria should strive to capture both the presentations of NPD (Fossati et al. 2005), while maintaining a focus on the different impairments in personality functioning. This is further supported by the node–network exploration, which confirms the centrality of the need for admiration criterion. In other words, mental health professionals perceive a criterion that describes both covert and overt functioning as a core element within the symptom network of NPD (Hermann et al. 2018). Indeed, while individuals with grandiose manifestations (characterised by agentic extraversion; Weiss et al. 2019) actively seek attention and admiration by talking about their successes, those with vulnerable manifestations (defined by higher neuroticism and negative emotionality (Weiss et al. 2019) tend to avoid standing out due to fear of humiliation, yet experience a hidden desire for recognition (Gabbard 2014). Despite choosing different strategies, both grandiose and vulnerable presentations of NPD may reflect a core need for external validation to maintain self‐esteem (Gabbard 1983). Therefore, the results suggest that the need for admiration is the central element influencing the entire symptomatic network of NPD, affecting both self and interpersonal functioning. This is in line with the psychodynamic perspective, suggesting that the core of pathological narcissism is a fragile and unstable underlying self‐esteem (Kernberg 1989; Kernberg 1998; Kohut 1968).

### 4.2 The Role of Antagonism Domain for NPD: a Confirmation

Within the NPD's domains network, the main result concerns the centrality of antagonism, confirming what is stated in Section III of the DSM‐5‐TR (APA 2013, 2022), where two pathological personalities under this domain are included: grandiosity believing and Attention seeking. This is consistent with previous research identifying antagonism as a core trait of narcissism (e.g., the trifurcated model of narcissism; Weiss et al. 2019). Indeed, existing evidence has shown that this dimension can be considered a unifying characteristic for overt and covert manifestations, with significant and positive correlations of both indices of grandiose and vulnerable narcissism (Crowe et al. 2019; Miller et al. 2016). More in‐depth, both forms share a common underlying feature of grandiosity believing. Indeed, overt narcissists display their grandiosity openly, seeking admiration and asserting their superiority, whereas covert narcissists conceal their grandiose self‐views behind a veil of insecurity and hypersensitivity (Miller et al. 2016; Pincus et al. 2014). Similarly, in both narcissistic manifestations, attention seeking is a fundamental characteristic, manifested through different behaviours and strategies. Specifically, overt narcissists actively seek the limelight and admiration, whereas covert narcissists subtly elicit attention through vulnerability and self‐pity (Atlas and Them 2008; Shane‐Simpson et al. 2020). From a node–node perspective, results showed that antagonism was also associated with the domains of detachment and negative affectivity. Indeed, although the DSM‐5 Section III (APA 2013, 2022) primarily focuses on the antagonism domain for NPD criteria, it acknowledges the potential for additional pathological personality traits from other domains to be specified to capture the broader spectrum of NPD presentations. For instance, the detachment trait, characteristic of the disinhibition domain, could be specified to describe the tendency to withdraw and inhibit emotions in the covert presentation (Zajenkowski and Szymaniak 2021). Additionally, research suggests that both grandiose and vulnerable presentations can exhibit features from the negative affectivity domain, including depression, anxiety (Huprich et al. 2012) and even hostility, which is common to both presentations (Czarna et al. 2021).

### 4.3 Practical Implications

The results of this research may have useful implications for clinical practice with patients with NPD. Firstly, identifying the core elements of this personality disorder may shed light on aspects that can most effectively support the diagnostic process (i.e., need for admiration and antagonism), highlighting the role of elements common to both grandiose and vulnerable presentations. Regarding treatment, the scientific literature highlights that NPD presents several challenges to clinicians (Weinberg and Ronningstam 2020). For instance, aspects like the lack of empathy and strong vulnerability to shame can make establishing a genuine alliance very difficult (see Caligor et al. 2015 for a review). From clinicians' perspectives, it can also be challenging to build rapport with NPD patients, who often make the therapist feel used, like a sounding board, or controlled, as if any cue could be a source of devaluation (Gabbard 2014). Understanding these difficulties and framing them as linked to the need for admiration to maintain self‐esteem can support and guide the clinician in the therapeutic process, also increasing their sense of expertise and effectiveness (Gori et al. 2022, 2023). Finally, the application of the network analysis approach (Borsboom and Cramer 2013) may guide treatment by also highlighting that working on core elements (i.e., need for admiration and antagonism) can serve as a gateway to intervening on other symptomatic aspects, improving both self and interpersonal functioning.

### 4.4 Limitations and Suggestions for Future Research

Although this study offers valuable insights into the network structure of NPD, it is crucial to acknowledge its limitations. First, the snowball sampling method employed for participant recruitment may have introduced selection biases. Future research may employ more robust sampling methods, such as random or stratified sampling, to ensure a more representative sample. Furthermore, only self‐report measures were used. Incorporating a multimethod approach (e.g., integrating the use of interviews) can be an interesting challenge for future research to provide more accurate findings. Finally, the use of clinician‐reported data without direct patient correspondence does not allow evaluating any differences between clinician perceptions and patient self‐reports. Integrating these two perspectives in future research could provide further enrichment and study data.

## 5 Conclusions

This study represents a pioneering effort to employ network analysis methodology to investigate the dynamics among the symptoms of NPD, from the perspective of mental health professionals. The findings underscore the centrality of the need for admiration among the criteria, and of antagonism among the domains. The present research also highlighted the possibility of recognising two groups of criteria: one linked to self‐functioning and the other to interpersonal functioning, with the need for admiration serving as a connecting element. These results offer valuable practical implications by enhancing existing research on NPD and improving clinical practice. The insights gained can support both diagnostic accuracy and the development of effective treatment strategies. By highlighting the interconnected nature of NPD symptoms, this research contributes to a more nuanced understanding of the disorder, ultimately aiming to improve patient outcomes through informed, evidence‐based approaches.

## Author Contributions

**Alessio Gori:** conceptualisation (lead); methodology (equal), formal analysis (equal), data curation (lead), writing – original draft preparation (equal), writing – review and editing (equal), supervision (lead). **Eleonora Topino:** methodology (equal), formal analysis (equal), data curation (equal), writing – original draft preparation (equal), writing – review and editing (equal).

## Funding

The authors have nothing to report.

## Ethics Statement

The research was approved by the Ethical Committee of the Integrated Psychodynamic Psychotherapy Institute.

## Consent

Informed consent was obtained by all the subjects involved in the research.

## Conflicts of Interest

The authors declare no conflicts of interest.

## Data Availability

The data that support the findings of this study are available from the corresponding author upon reasonable request.
